# Supplementary material for: Differing terminology used to describe antimicrobial resistance can influence comprehension and subsequent behavioural intent
Source: Commun Med (Lond). 2025 Apr 29;5:146. doi: 10.1038/s43856-025-00849-z (PMC12041392; doi:10.1038/s43856-025-00849-z)
Supplement: Supplementary file 2 — Supplementary Information [file 43856_2025_849_MOESM2_ESM.pdf]

<u>Supplementary Method – Participant flow through RCT & Survey Questions</u>

<u>Introduction:</u>
Welcome and thank you for participating in this survey.
Task: In this exercise, we are going to show you some information about antibiotics and when to use them. You can look at this information for as long as you like. We are then going to ask you some questions about the information you saw.
Duration: The survey should take about 5 minutes to complete and requires your attention, so please only participate if you can dedicate this time!
Withdrawal: You can withdraw your participation at any point during the survey by clicking 'Exit and clear survey' in the top right corner.
Please note that you cannot go back to previous pages.
[Participants provided with online Participant Information Sheet]
<u>Attention Check</u>
[AttCheck1] People are very busy these days and many do not have time to pay close attention to what they are reading. We are testing whether people read questions. To show that you've read this much, answer both "Moderately interested" & "Slightly interested".

1. Extremely interested
2. Very interested
3. Moderately interested
4. Slightly interested
5. Not interested at all

[new screen]
[AttCheck2, If AttCheck1 answered = "Extremely interested", "Very interested" or "Not interested at all"] You didn't select the correct answers to our last question. Your attention to the survey questions is very important for our research, so we'd like to give you another chance to respond. To show that you are paying attention, answer both "Extremely interested" and "Very interested."

1. Extremely interested
2. Very interested
3. Moderately interested
4. Slightly interested
5. Not interested at all

[new screen]
Participants randomly assigned to see one of the four messages – "Antimicrobial Resistance – AMR", "Superbugs", "Antibiotic Resistance" or "The Antibiotic Crisis".

[new screen]

<u>Recall</u>
[recallTerm] Which of the following terms was at the top of the poster?

- Antimicrobial Resistance "AMR" (correct if arm "Antimicrobial Resistance – AMR")
- Superbugs (correct if arm "Superbugs")
- Antibiotic Resistance (correct if arm "Antibiotic Resistance")

- The Antibiotic Crisis (correct if arm “The Antibiotic Crisis”)
- Drug-resistant infections

[recallContent] What was the poster about?

- The issue of infections becoming resistant to antibiotics because of overuse of antibiotics.
- The risks of negative side-effects when people take antibiotics
- The benefits of natural or homeopathic remedies.
- A new virus that may spread globally.
- Don’t know

[recallFreeText] Please give a brief definition of the following term: Superbug / Antibiotic Resistance / Antibiotic crisis / AMR.

Free text response

[new page]

<u>Comprehension</u>

[New screen. Show image again]

[compDefinition] Which of the following definitions best describes Superbugs / Antibiotic Resistance / Antibiotic crisis / AMR?

- Infections have become resistant to antibiotics
- Our bodies are becoming increasingly immune to infections
- Our bodies are becoming resistant to antibiotics
- A global shortage of antibiotics
- Our bodies are becoming better at tolerating side-effects of antibiotics due to frequent use

[compCause] What has caused the issue of Superbugs / Antibiotic Resistance / Antibiotic crisis / AMR

- Overuse of antibiotics
- Overuse of hand-sanitizer
- No discovery of new antibiotics
- Changes in bacteria
- Overconsumption of supplements like probiotics
- Natural changes to our immune system over time

[compSolution] What is the solution to tackling Superbugs / Antibiotic Resistance / Antibiotic crisis / AMR?

- Only using antibiotics when they are absolutely necessary
- Encouraging natural immunity through exposure to germs
- Implementing global bans on all antibiotic usage
- Increasing the dosage of antibiotics to overcome resistance
- New drug discovery

[confidence] Please rate how confident you are in your responses.

- Extremely confident
- Very confident
- Somewhat confident
- Not confident

Intent

[new screen. Show image again]

[intent] Imagine that you are unwell with a mild sore throat, a cough, a runny nose, and slight fatigue. How likely are you to...

- Visit your GP?
- Visit a pharmacist for advice?
- Request antibiotics from a doctor?
- Take medication (other than antibiotics)?

[Not at all / Somewhat likely / Moderately likely / Very likely]

Intent 2

[new screen. Show poster again]

[intent_stop if intent_antibiotics == "Y"] You previously said you would request antibiotics if you were feeling unwell. Now imagine you are feeling better before you finish the course of antibiotics. How likely are you to stop the antibiotic course early?

[Not at all / Somewhat likely / Moderately likely / Very likely]

Attitudes towards AMR

[attitudesContent] To what extent do you agree with the following statements:

- Superbugs / Antibiotic Resistance / the Antibiotic crisis / Antimicrobial Resistance (AMR) poses a risk to global health.
- It is important to address Superbugs / Antibiotic Resistance / the Antibiotic crisis / Antimicrobial Resistance (AMR).
- Superbugs / Antibiotic Resistance / the Antibiotic crisis / Antimicrobial Resistance (AMR) is/are an urgent issue.
- I understand what Superbugs / Antibiotic Resistance / the Antibiotic crisis / Antimicrobial Resistance (AMR) means.
- Superbugs / Antibiotic Resistance / the Antibiotic crisis / Antimicrobial Resistance (AMR) is an issue that could have an impact on my own health.

[Not at all / A little / Moderately / Very much]

[new page]

Additional Questions

[familiar] Which of the following terms are you familiar with? *Please select all that apply.*

- -**[AMR, if treatment != 1]** Antimicrobial Resistance "AMR"
- - **[bugs, if treatment != 2]** Superbugs
- - **[AR, if treatment != 3]** Antibiotic Resistance
- - **[AC, if treatment != 4]** The Antibiotic Crisis
- - **[nota]** None of the above

[treatment != 1] AMRFreeText]

Please give a brief description of what you think Antimicrobial Resistance (AMR) is? Free text response

Additional Demographics

[antibioUse] Have you taken antibiotics in the past year?

- Yes
- No
- Not sure

[antibioHousehold] Has anyone in your household taken antibiotics in the past year?

- Yes
- No
- Not sure

[healthcarePro] Do you work in the healthcare industry?

- Yes
- No

[nativeLang] Is English your first language?

- Yes
- No

[healthcareProType, if healthcarePro == "Yes"] Does your job involve prescribing antibiotics?

- Yes
- No
