# Supplementary material for: Differing terminology used to describe antimicrobial resistance can influence comprehension and subsequent behavioural intent
Source: Commun Med (Lond). 2025 Apr 29;5:146. doi: 10.1038/s43856-025-00849-z (PMC12041392; doi:10.1038/s43856-025-00849-z)
Supplement: Supplementary file 3 — Description of Additional Supplementary Files [file 43856_2025_849_MOESM3_ESM.pdf]

Description of Additional Supplementary Files

File name: Supplementary Data
Description: Full logistic regression models
